# Comparative Analysis of Ultrasonic NDT Techniques for the Detection and Characterisation of Hydrogen-Induced Cracking

**DOI:** 10.3390/ma15134551

**Published:** 2022-06-28

**Authors:** Rymantas J. Kažys, Liudas Mažeika, Vykintas Samaitis, Reimondas Šliteris, Peter Merck, Žydrius Viliūnas

**Affiliations:** 1 Prof. K. Baršauskas Ultrasound Research Institute, Kaunas University of Technology, K. Baršausko St. 59, LT-51423 Kaunas, Lithuania; rymantas.kazys@ktu.lt (R.J.K.); liudas.mazeika@ktu.lt (L.M.); remondas.sliteris@ktu.lt (R.Š.); 2 Dekra Industrial AB, Box 13007, 402 51 Gothenburg, Sweden; peter.merck@dekra.com; 3 Dekra Industrial, Taikos Av. 7, LT-31107 Visaginas, Lithuania; zydrius.viliunas@dekra.com

**Keywords:** hydrogen induced cracking, high temperature hydrogen attack, ultrasound, non-destructive testing, material characterisation

## Abstract

The article is devoted to the investigation of ultrasonic inspection techniques suitable for detecting hydrogen-induced cracking (HIC) and a high-temperature hydrogen attack (HTHA), which are of great importance in petrochemical and refinery industries. Four techniques were investigated: total focusing method (TFM), advanced velocity ratio (AVR) measurement, advanced ultrasonic backscatter technique (AUBT) and time of flight diffraction method using ultra low angle ultrasonic transducers (TULA). The experimental investigation has been carried out on two carbon steel samples cut off from a heat exchanger of an oil refinery and potentially affected by HIC. It was shown that the AVR technique did not reveal any damage and was not effective in the case of the investigated samples due to a thin damage zone with respect to the total thickness of the samples. The AUBT method enabled us to indicate and classify the presence of the hydrogen-induced damage; however, it is complicated to use in practise due to the need perform measurements exactly at the same position using two transducers of different frequencies. The method is more suitable for the verification of damage at a particular position, rather than for scanning. Both other methods—TFM and TULA—enabled us to identify the presence of HIC in large areas of samples. The obtained results have been verified using a metallographic analysis of the section cut from the side of the sample. The results of metallographic examinations have been compared with indications observed using above mentioned techniques and a good correspondence was obtained. It was demonstrated, that the TFM method can detect cracks with dimensions close to 200 µm, while larger cracks of 2 mm were observed very evidently using a 7.5 MHz phased array. Overall, the results suggested that the TULA method is the most suitable method for the primary detection of hydrogen-induced cracking, while the TFM is recommended for the precise assessment of the extent of the detected cracking.

## 1. Introduction

Pipelines and pressure vessels carrying hazardous substances in petrochemical and refinery industries are subjected to hydrogen-rich environments and thereby suffer from progressive structural degradation called hydrogen-induced cracking [1]. According to the internal pressure theory, atomic hydrogen is absorbed on the surface of the steel and diffuses inside the lattice in the form of protons [2,3,4]. When diffused into the metal, atomic hydrogen accumulates at interstitial locations–voids, grain boundaries, dislocations, and manganese sulphide inclusions [5,6]. At such trap sites, hydrogen recombines to a molecular form, creating high internal pressure in the immediate vicinity. Depending on the temperature of the environment, the main two types of damage can be distinguished: hydrogen-induced cracking (HIC), which occurs at room temperature, and a high temperature hydrogen attack (HTHA), which occurs at temperatures above 200 °C.

In sour environments such as oil or acid gases, corrosion of carbon and low alloy steels may start due to the influence of atomic hydrogen. Such corrosion leads to embrittlement of steel constructions [7,8]. Atomic hydrogen originates during the chemical reaction of hydrogen sulphide H2S, which is present in crude oil with water [7]. After some time, atomic hydrogen becomes an ion, which, due to smaller dimensions, moves into steel and accumulates in internal cavities [9]. This hydrogen creates pressure inside the cavities and may cause defects such as laminations or cracks. The type of appearing defects depends on how the steel can sustain plastic deformation under tensile stress before failure. 

In the case of ductile steels, the developed cracking is called hydrogen-induced cracking (HIC) [10]. Hydrogen-induced cracking appears in the unstressed zones of a bulk material. Once the cracks are established, they can incorporate even more hydrogen, leading to successive crack growth and connection in a stepwise appearance. The growth of the established internal cracks is known to be assisted by weakened interatomic bonds in the host metal, increased dislocation mobility and promoted vacancy formation [11].

In cases where the steel is not ductile, for example, the high strength steel or heat affected zones close to a weld, multiple cracks in a base metal join each other, producing a through-thickness crack which is perpendicular to the surface of the steel wall. Such cracking is produced by high stress and is therefore called Stress Oriented Hydrogen-Induced Cracking (SOHIC) [8]. SOHIC starts from the hydrogen-induced cracking and later, due to the stress, links separate laminations in a through the wall thickness cracks. Such surface breaking cracks are considered the most dangerous.

At temperatures above 200 °C, HTHA takes place, which is assisted by high temperature and pressure. It is associated with carbon or low alloy steel, where diffused hydrogen in contact with dissolved carbon at temperatures above 200 °C promotes internal decarburization of the structure, producing methane gas [5,12]. As methane molecules are too large to diffuse out of material, they build-up internal gas cavities with pressures up to two orders of magnitude higher than that of hydrogen itself [13]. When the pressure exceeds the fracture toughness of the material, the resulting gas-filled cavities cause the plastic deformation of the surrounding lattice, thereby forming internal microcracks [11]. Eventually, the microcracks coalesce to fissures and blisters, compromising the structural integrity of the pipeline networks.

Hydrogen cracking is an internal damage that develops for a while before it can be detected. Early attempts to mitigate the risks of hydrogen damage established the so-called Nelson curves that specify safe operational conditions in terms of temperature and hydrogen pressure for different types of steel [14]. However, early failures reported with C-0.5Mo steel [15] and a quite recent accident where HTHA caused carbon steel heat exchanger failure at the Tesoro Anacores refinery in 2010 [16] raised significant concern about the reliability of the Nelson curves. In fact, new Nelson curves for non-welded PWHT (post weld heat treatment) and welded non-PWHT carbon steel were established in 2016, yielding a significant amount of existing piping to operate above the “safe” zone [17]. 

Hydrogen damage is a complex phenomenon that, in addition to temperature and hydrogen pressure, is a function of carbide stability, applied/residual stress, grain size, type of weld and time in service [16,18]. Given that for most in-service assets the abovementioned parameters such as grain size or carbide stability cannot be defined owing to the mechanical, physical and chemical nature, early stage hydrogen damage detection is of vital importance to ensure mechanical integrity and prevent equipment damage. The successful identification of HTHA at the infantile stage is highly dependent on the non-destructive testing (NDT) techniques employed.

Different ultrasonic techniques are frequently used as a primary tool for determining the extent of hydrogen damage, namely, acoustic emission, non-linear ultrasonic testing, Crack Indication Density (CID) method, tri-lateral phased array scanning, advanced velocity ratio (AVR), advanced ultrasonic backscatter technique (AUBT), time of flight diffraction (TOFD), TULA (TOFD ultra low angle) and total focusing method (TFM) [17,19,20,21,22,23,24,25,26,27]. 

Currently, API RP 941:2016 accepts AVR, classic TOFD and AUBT techniques for the in-field inspection of HTHA. However, it clearly states that the velocity ratio technique should be used for advanced stage HTHA detection, pattern recognition and frequency dependence AUBT are recommended as complimentary or in set with other techniques, while TOFD can be used to detect developed cracks rather than HTHA fissures [28]. Other techniques, such as CID and tri-lateral phased array scanning are more associated with cold HIC, while newly developed approaches such as TULA and TFM have not been widely explored.

This introduces some confusion as non-destructive techniques applied for detection of defects caused by HIC or HTHA are rather different. On the other hand, it is not always known, a priori, what kind of impact hydrogen has in particular cases. This means that for practical purposes, it would be useful to have universal non-destructive techniques suitable both for detecting the damage caused by HIC and HTHA. In this paper, we investigate, experimentally, the detection capabilities of defects caused by HIC, applying techniques used in the case of HTHA. Experimental investigations were carried out using steel samples affected by HIC.

Therefore, the objective of this work was to compare common non-destructive testing techniques for the detection of HIC on samples and select the most suitable method for use in practical cases. For this purpose, we selected AVR, AUBT, TULA and TFM as the methods that can provide best outcome.

## 2. Overview of the Selected Hydrogen Damage Detection Techniques

Microcracks and the successive build-up of methane molecules at grain boundaries eventually change the Young and shear modulus of the structure. Hasegawa proposed measuring the velocity ratio between the longitudinal and shear ultrasonic waves that are directly dependent on the elastic properties of the medium using the advanced velocity ratio AVR technique [29]. It was found that the presence of microcracks changes longitudinal and shear velocities differently, the former being more affected by discontinuities. As a result, any increase in velocity ratio above 0.55 should indicate the presence of HTHA, while an obtained ratio value of 0.59 will show advanced stages of HTHA. Owing the relatively small changes in velocity ratio, the API RP 941:2016 recommends the velocity ratio technique for inspecting the parent metal in cases where at least 10% of the structure has been damaged [28]. Given the limitations of coupling, between shear wave transducer and the sample, the AVR technique is considered as manual and eventually can miss the signatures of the early stages HTHA, which are highly localised. The advanced ultrasonic backscatter technique AUBT exploits the scattering of ultrasonic waves at fissures induced by HTHA. Multiple measures can be used to evaluate the backscatter, namely amplitude, pattern recognition and frequency dependence [24]. While amplitude backscatter evaluates the magnitude of noise content, pattern recognition and frequency dependence techniques exploit the attenuation of the backscattered noise. Wang found that the amplitude of the backscattered noise rises at the first arrival of the backscattered signal and then decreases exponentially due to attenuation when the wave travels further through the damaged region [30]. The frequency dependence of such a phenomenon was perceived for HTHA-induced defects, showing the appearance of the elongated tail of the backscatter response at lower inspection frequencies as the attenuation term reduced. Such a phenomenon allowed us to distinguish between the HTHA and non-HTHA noise caused by inclusions, as the tail elongation was solely related to HTHA [31]. Another in field accepted HTHA detection technique—TOFD—implements the detection of diffracted waves produced by HTHA crack tips with a set of angle beam transmitting and receiving probes facing each other. The positioning of the detected indications is based on time-of-flight analysis; hence, it allows quite an accurate assessment of defect size and location [32]. The limitations reported with TOFD are related to existing blind zones due to the lateral wave and backwall echo, which shadow diffracted waves near the front and back surfaces. Hence, the TOFD technique is reported to be effective for sufficiently penetrating HTHA detection [24]. Recent advances in TOFD technology introduced TULA probes (TOFD Ultra Low Angle) that consist of two acoustically isolated piezoelectric elements producing low refraction angles [27]. A performance evaluation study on blind flange and parent metal samples demonstrated the robustness of TULA probe in distinguishing HTHA from the localised inclusions. The investigation, supported by metallographic analysis, demonstrated that in the presence of inclusions, TULA and TFM can correctly identify the defect type, while the AVR and AUBT techniques can identify other types of damage such as HTHA [27]. Similarly, in the case of HTHA, TULA correctly identified the damage, while the AVR technique was the only one which gave false negative results among all other investigated NDT techniques. Although the TULA technique has been designed with HTHA detection in mind, to date, it has not been widely applied, either in the field, or in the research community. Another quite recent HTHA detection technique is based on the application of ultrasonic phased arrays. The first attempts to use ultrasonic phased arrays for HTHA detection were demonstrated by Birring, who showed the typical responses of HTHA and stingers [19]. With the advent of the total focusing method, high resolution crack detection became possible and was demonstrated by several authors [33,34].

## 3. Test Samples and Experimental Techniques

In order to elucidate the HIC detection capacity of each technique discussed above, experimental investigations were carried out on the set of samples. In this study, two carbon steel samples cut off from a heat exchanger of an oil refinery and potentially affected by HIC were investigated. A total of 5 samples were investigated; however, in the article, we present the results obtained on two—No.4 and No.5—which show the most typical indications of HIC. The dimensions of the samples were as follows:

- Sample No. 4: 540 × 567 × 46 mm^3^;
- Sample No. 5: 425 × 535 × 46 mm^3^.

**Figure 1:**
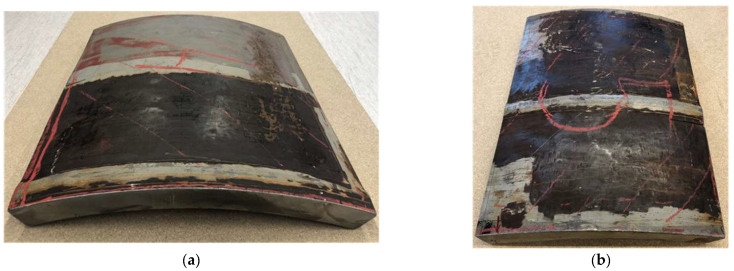
Segments of heat exchanger pipe used for comparison of HTHA detection algorithms: (**a**) sample no. 4 and (**b**) sample no. 5.

Both samples contained a circumferential weld, as can be seen from Figure 1. Among the abovementioned samples, Sample No. 4 was used for the comparison of HIC inspection techniques—AVR, AUBT, TULA and TFM—while Sample No. 5 was analysed with TULA and TFM only; the results obtained on this sample were, however, supported with a metallographic analysis.

Chemical composition of sample no. 4, measured with an Olympus Vanta XRF spectrometer, is presented in Table 1.

**Figure 2:**
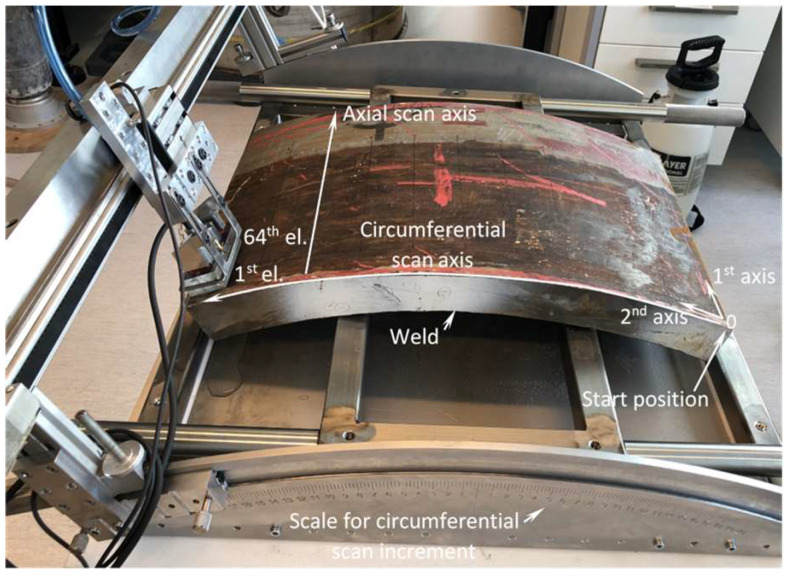
Experimental set-up for full surface TFM data acquisition.

To acquire data for the analysis, the entire surface of both mock-ups was scanned with phased array using the full matrix capture (FMC) approach and then the internal volume of the structure was reconstructed using TFM. As a result, a TFM cartography of both samples was obtained. While other available techniques, such as AVR and AUBT, are more localised according to their nature, measurements using the AVR and AUBT techniques were taken at discrete locations and subsequently compared with TFM cartography data. The TFM scan resolution along the axial direction was set to 1 mm, while the scanner increment at circumferential direction was fixed at 3 mm. The axial scan was performed using a single axis belt scanner with an encoder resolution of 16 pts/mm. The circumferential scan increment was manual according to the prepared scale on the test piece holder, where 0.25° corresponded to a 3 mm position shift (see Figure 2). The axial and circumferential scan distances were different and determined by the physical dimensions of each test piece. They are summarised in Table 2. 

The defined axial and circumferential scanning positions established a measurement grid, so each measurement using AUBT, TULA or AVR techniques at discrete positions could be compared both to each other and to TFM. The experimental set-up for full surface TFM scan acquisition is presented in Figure 2.

## 4. Experimental Evaluation of Ultrasonic NDT Techniques for Detection of HTHA

In this section, HIC detection capacity between the TFM, AVR, AUBT and TULA techniques will be compared on sample no. 4. The section starts with the acquisition of TFM cartography data, which are used as the reference for other techniques. The section continues with AVR, AUBT and TULA measurements and a comparison with TFM reconstruction at the same location. Finally, the section summarizes the findings on each technique.

### 4.1. The Total Focusing Method (TFM)

The Total Focusing Method (TFM) is an ultrasonic array technique which is used to synthetically focus an ultrasonic beam at every point on a region of interest. The data acquisition is performed using the FMC approach, which means that each element of the transmitting array is excited one by one. The signals received by all array elements after each shot are recorded and the full matrix of the received ultrasonic signals is stored in a memory. After that, by means of signal processing, the ultrasonic wave is focused on every point of interest and a high-resolution image is obtained.

**Figure 3:**
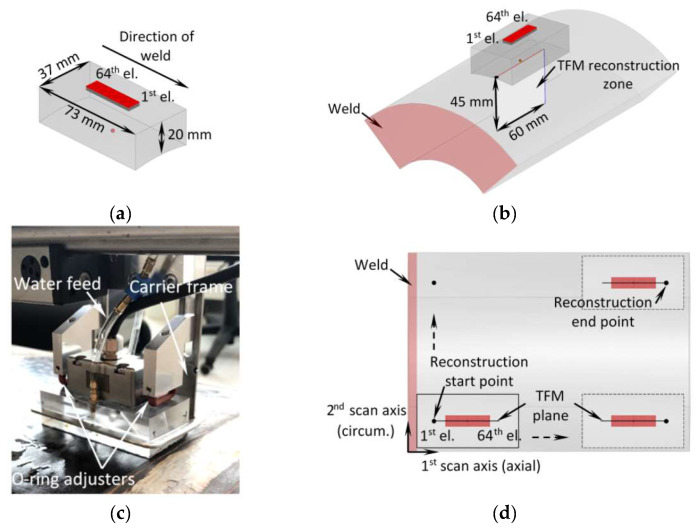
(**a**) Array positioning and dimensions of the wedge; (**b**) array positioning on pipe segment and dimensions of TFM reconstruction zone; (**c**) phased array holder in the experimental stand; (**d**) scanning sequence.

To obtain TFM cartography data, the measurements were taken using Eddyfi Gekko PAUT system and 7.5 MHz 64 element phased array (Imasonic 64L7.5-G3). The array had 0.5 mm pitch, interelement spacing of 0.1 mm and active area of 31.9 mm × 9 mm. The bandwidth of array was approximately 55% at −6 dB. The cylindrically concave Plexiglas wedge with dimensions of 73 mm × 37 mm × 20 mm and a longitudinal wave velocity of 2700 m/s was used as an interface between the test piece and the array. To ensure proper acoustic contact, the water supply system and special O-ring adjusters were used (see Figure 3c). The array and the wedge configuration are shown in Figure 3a,c.

The array was driven with 100 V bipolar square pulse, and the signals were sampled with 16bit ADC at 100 MHz frequency. The TFM reconstruction was performed using LL wave mode. At each increment, the TFM reconstruction zone was 60 mm wide and 45 mm high, as illustrated in Figure 3b. Each TFM reconstruction plane had 94k points, which corresponds to a pixel size of 0.17 mm (λ/46). In each experiment, the 1st element of the array faced the weld, as shown in Figure 3d. Throughout the experiments, the 1st scan axis corresponded to the axial pipe direction, while the second scan axis to the circumferential direction (see Figure 3d). The main parameters used in the experiment are summarised in Table 3. Each TFM image largely overlapped with the previous one, as the 1st scan axis coincided with the axis of the phased array and the scanning was performed with the step 1 mm. So, to improve the signal to noise ratio (SNR), the final B-scan image along the first scan axis was obtained by combining TFM images at all scanning positions and averaging overlapping image points. The averaged B scan images afterwards were combined with other ones obtained at different scan position along the second axis into the 3D TFM data set, which can be analysed in different cross-sections.

**Figure 4:**
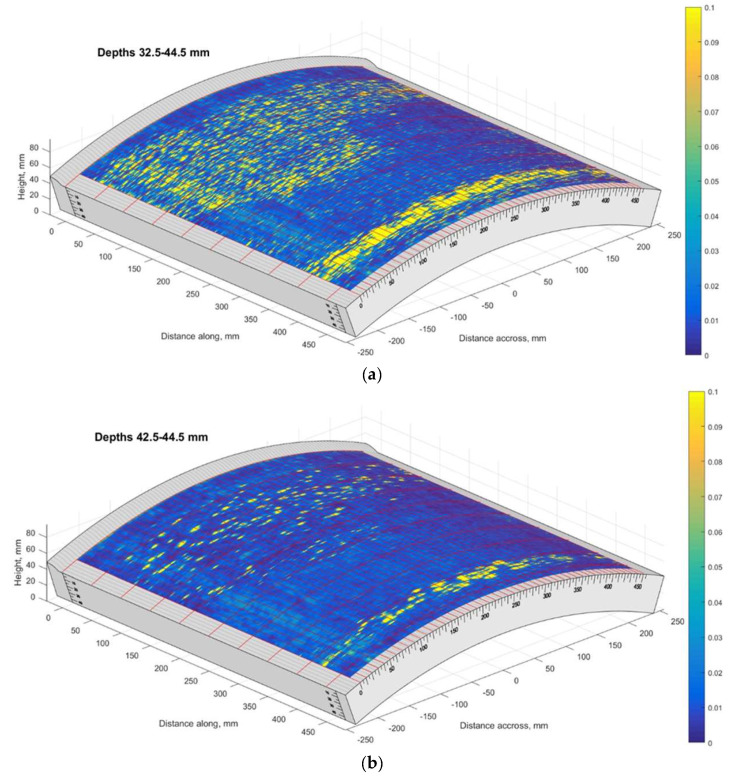
The TFM scan of the Sample No. 4: (**a**) depth range of 32.5–44.5 mm; (**b**) depth range of 42.5–44.5 mm. Vertical colour bar represents the peak-to-peak amplitude in arbitrary units.

The TFM C-scan results on sample No. 4 are presented in Figure 4. The first part of the figure (Figure 4a) shows the aggregated TFM response over the depth range of 32.5–44.5 mm. The second part of the figure (Figure 4b) presents the likely indications of subsurface blisters (depth range of 42.5–44.5 mm). Here, 44.5 mm corresponds to the overall thickness of the mock-up No. 4.

**Figure 5:**
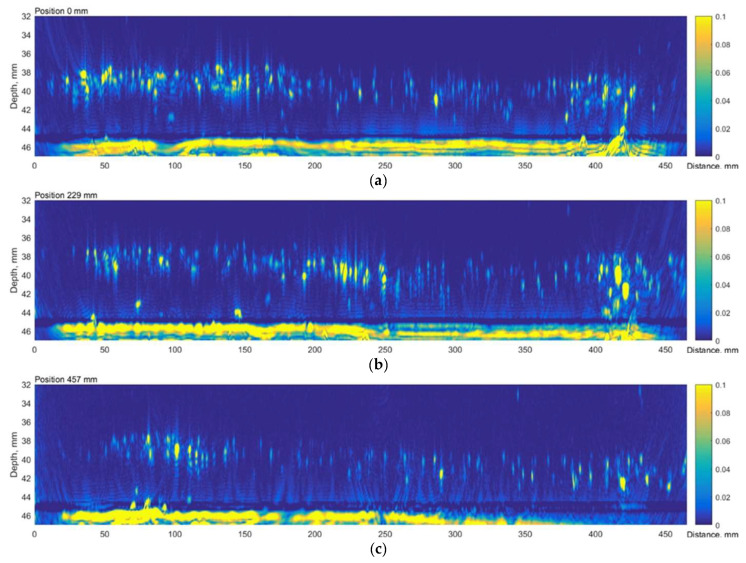
The axial scan B scan images of the TFM reconstruction: (**a**) at 0 mm; (**b**) 229 mm and (**c**) 457 mm circumferential locations. Vertical colour bar represents the peak-to-peak amplitude in arbitrary units.

The results presented above suggest that the HIC damage is distributed non-uniformly. There is a wide non-regular zone close to the weld and an extensive, concentrated region around the circumference 400 mm away from the weld. The TFM B-scan images corresponding to the first (0 mm), middle (229 mm) and the last (457 mm) circumferential scan positions are presented in Figure 5. The results clearly indicate concentrated reflectors, likely voids and cracks, that could be a result of HIC damage at approximately 40 mm depth or 5 mm above the backwall.

### 4.2. Advanced Velocity Ratio (AVR) Measurement

The AVR technique is based on the measurement of the ratio of shear and longitudinal wave velocities in damaged and undamaged zones. The main problem is to measure ultrasound velocities, for which it is necessary to know or to measure the object’s local thickness, which, in the case of in situ inspections, is impractical and usually even impossible. Instead of absolute velocity measurement, the AVR technique estimates the time-of-flight ratio between the longitudinal and shear wave, which is dimensionless and independent of the object thickness. If this ratio is close to 0.55 in different areas of the object, the material is considered to be unaffected by HIC. In contrast, if such a ratio becomes higher than 0.55, then this part of the sample under a test is likely to be affected by HIC. The effectiveness of the AVR method essentially depends on the HIC penetration depth. The subsurface defects will have a minor influence on wave velocity, hence AVR is recommended for inspection when at least 10% of the structure has been affected by HIC.

To investigate HIC detection capacity, the AVR technique was explored on sample no. 4. The measurements were performed with the medium frequency ultrasonic system ULTRALAB (Ultrasound Institute, Kaunas University of Technology, Lithuania). For generation and reception of longitudinal and shear waves, the Panametrics-NDT Olympus 5MHz transducers (V109, 5 MHz/0.5″ and V155, 5 MHz/0.5″) were used. The excitation voltage was 20 V and the total system gain −42 dB. Both transducers were placed at selected locations on the surface of sample no. 4. In total, five locations were investigated. The positions of each AVR measurement location are summarised in Table 4.

**Figure 6:**
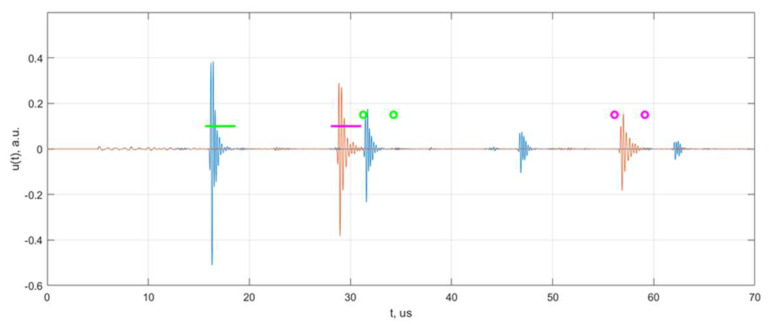
The measured signals of longitudinal (blue) and shear (red) waves. Horizontal lines indicate the time windows for the first backwall reflection, the circle markers show time gates for the second backwall reflection.

The propagation times of longitudinal and shear waves were measured using a cross-correlation method. The example of longitudinal and shear wave signals obtained at the first measurement location is presented in Figure 6.

The times of flight (ToF) of longitudinal and shear wave using the cross-correlation technique were measured using two methods:

1. Using the reference signal;
2. By measuring the time difference between the first and the second reflections.

The advantage of the first approach is that the first bottom reflection is always clearly visible. However, calibration is required to assess a systematic correction of the method. The second approach does not require calibration, although it is not applicable in the absence of the second backwall reflection.

According to the first approach, the propagation time of longitudinal and shear waves were calculated in the following steps:

1. Two windows in the time domain are selected for the first reflection of the longitudinal wave [*t_L_*_1_, *t_L_*_2_] and the shear wave [*t_S_*_1_, *t_S_*_2_];
2. The delay time of the longitudinal and shear waves signals with respect to the reference signals are calculated using the cross-correlation method:

(1) $${\begin{array}{l} t_{Lcc}=\arg\left\{\underset{t∍\left[t_{L1}÷t_{L2}\right]}{\max}\left[corr\left[u_{L}\left(t\right),u_{ref}\left(t\right)\right]\right]\right\}\\ t_{Scc}=\arg\left\{\underset{t∍\left[t_{S1}÷t_{S2}\right]}{\max}\left[corr\left[u_{S}\left(t\right),u_{ref}\left(t\right)\right]\right]\right\} \end{array}}^{},$$

where by “corr” denotes the cross-correlation function; *u_ref_*(*t*) is the reference signal.

3. The absolute propagation times of both waves are estimated:

(2) $$\begin{array}{l} t_{LW}=t_{Lcc}+t_{L1}\Delta t_{ref}\\ t_{SW}=t_{Wcc}+t_{S1}\Delta t_{ref} \end{array},$$

where Δ*t_ref_* is the correction parameter determined for a particular reference signal during calibration. The reference signal was obtained using V2 calibration block.

According to the second approach, the time differences between the first and second back-wall reflections were determined as cross-correlation lag. 

**Figure 7:**
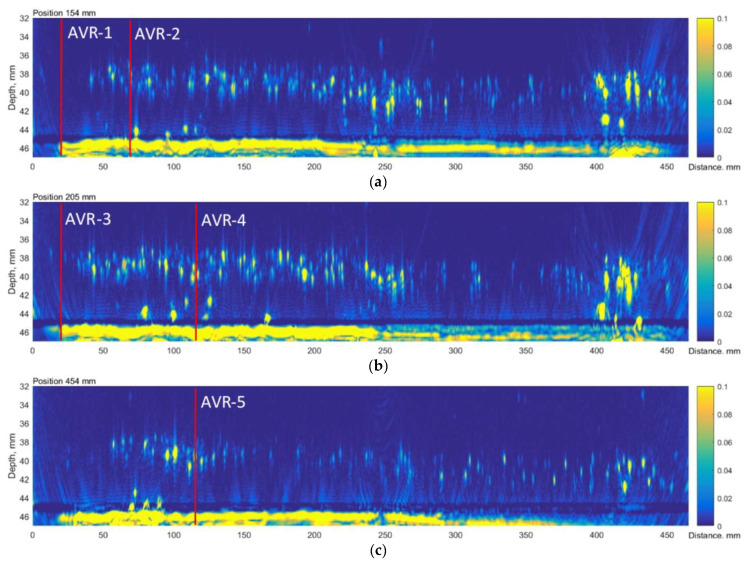
TFM reconstructions at different circumferential locations, indicating positions of AVR measurements: (**a**) 1st and 2nd measurement points, (**b**) 3rd and 4th measurement points, (**c**) 5th measurement point. Vertical colour bar represents the peak-to-peak amplitude in arbitrary units.

The obtained velocity ratio is equal 0.548 ± 0.01 and does not depend on the measurement position, what is illustrated by TFM data at Figure 7. According to this method it is less than 0.55, thus it does not indicate the presence of HIC at these positions in the sample. However, at the second, fourth and fifth positions, some reflectors can be observed in the TFM image, likely due to HIC damage (see Figure 7). It can be stated also that indications are observed at 8mm above the back wall, so it can be assumed that the damage is present at only 18% of total thickness, so it is completely understandable that the influence of the damage on the propagation time will be minor. So, it can be concluded that the AVR method possess poor sensitivity for the detection of HIC at a depth close to the bottom, at least in the case of thick samples.

### 4.3. The Advanced Ultrasonic Backscatter Technique (AUBT)

The advanced ultrasonic backscatter technique (AUBT) is based on the analysis of the ultrasonic signals backscattered by fissures and is used to identify the presence of micro-cracking in a parent material. The method enables the early recognition of potential HTHA sites. There are a few different versions of this technique and most advanced is pattern recognition and frequency dependence methods.

The pattern recognition technique is based on a phenomenon where the signal backscattered by HTHA region rises at the beginning of this zone and after that rapidly decreases due to the additional attenuation of the signal backscattered by fissures located further from the ultrasonic transducer. This phenomenon depends on a frequency of the ultrasonic wave. As a result, the backscattered noise after the first reflection from fissures is attenuated more at higher frequencies. The AUBT technique compares the backscattered noise in the signals measured at the same point using lower and higher frequency transducers. The observation of relatively lower noise levels after the first reflection in the case of higher frequency measurements indicates the presence of the HTHA.

An investigation of this method was performed on sample No. 4. For measurements, ultrasonic system ULTRALAB and two Panametrics-NDT Olympus ultrasonic transducers of 5MHz (V126, 5 MHz/0.3755″) and 20MHz (V116, 20 MHz/0.125″) were used.

According to the AUBT, the measurements should be carried out using two frequencies exactly at the same position. So, such a method is suitable only for the assessment of the presence of HTHA or HIC only at a discrete location. In total, five measurement locations were selected for AUBT measurements, as summarised in Table 5. Slightly different, compared to AVR method, locations have been selected intentionally, as in this case the particular waveform of indications is required, which possesses not only a single reflection, but also a sufficient level of structural noise behind the first reflection.

**Figure 8:**
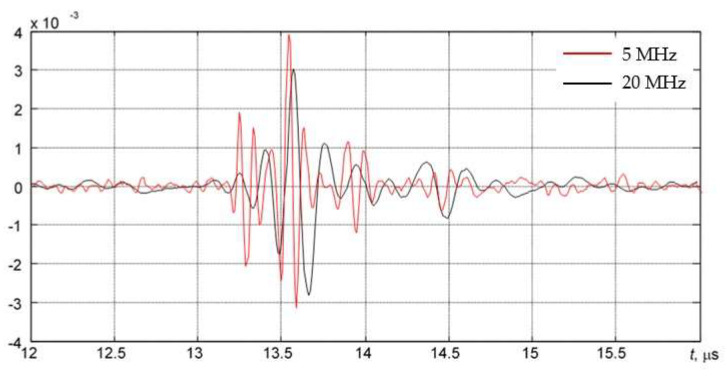
The waveforms of the received signals at the point AUBT-1 with 5 MHz (black) and 20 MHz (red) transducers.

**Figure 9:**
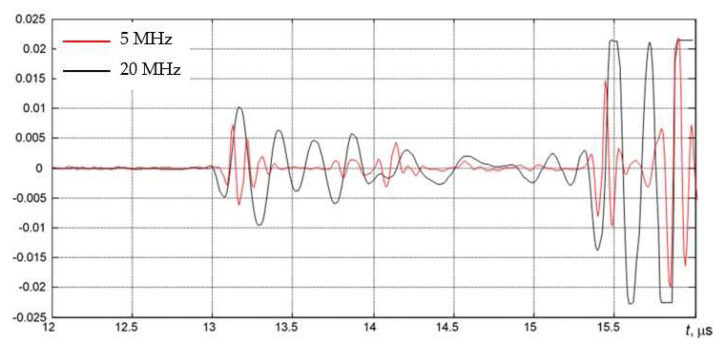
The waveforms of the received signals at the point AUBT-2 with 5 MHz (black) and 20 MHz (red) transducers.

**Figure 10:**
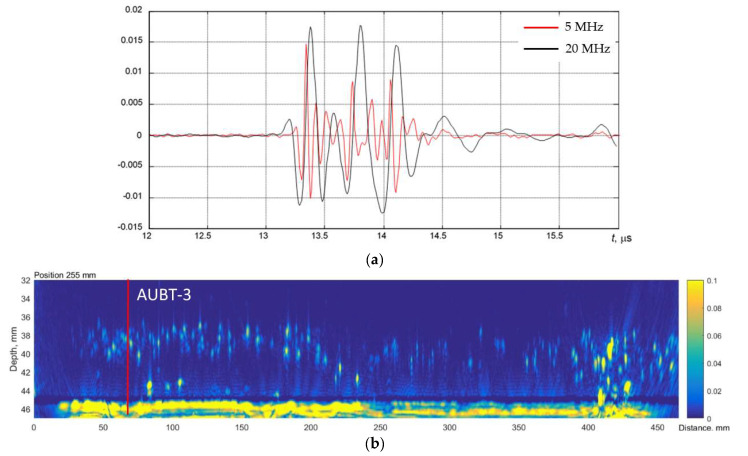
(**a**) The waveforms of the received signals at the point AUBT-3 with 5 MHz (black) and 20 MHz (red) transducers and (**b**) the corresponding TFM image, showing appropriate AUBT measurement location. Vertical colour bar represents the peak-to-peak amplitude in arbitrary units.

**Figure 11:**
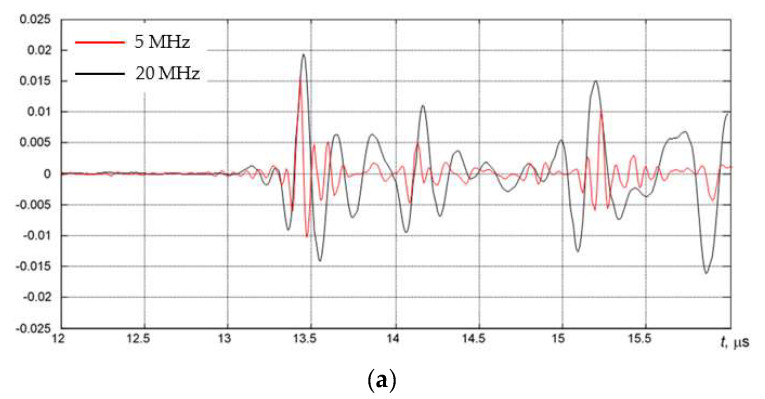

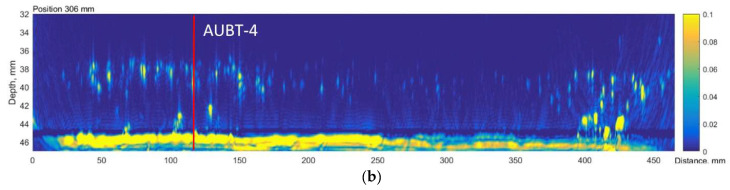
(**a**) The waveforms of the received signals at the point AUBT-4 with 5 MHz (black) and 20 MHz (red) transducers and (**b**) the corresponding TFM image, showing appropriate AUBT measurement location. Vertical colour bar represents the peak-to-peak amplitude in arbitrary units.

**Figure 12:**
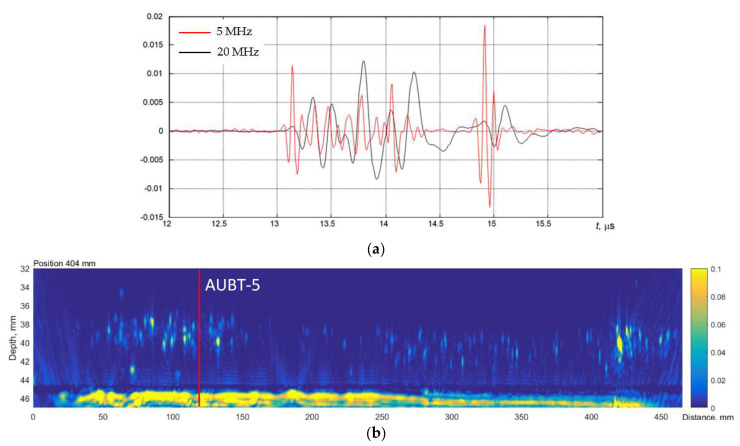
(**a**) The waveforms of the received signals at the point AUBT-5 with 5 MHz (black) and 20 MHz (red) transducers and (**b**) the corresponding TFM image, showing the appropriate AUBT measurement location. Vertical colour bar represents the peak-to-peak amplitude in arbitrary units.

The AUBT-1 and AUBT-2 locations coincide with the AVR measurements at AVR-1 and AVR-4. The AUBT-3, AUBT-4, and AUBT-5 are acquired at different locations than AVR. The results of AUBT measurements are presented in Figure 8, Figure 9, Figure 10, Figure 11 and Figure 12. The figures show waveforms of ultrasonic signals, obtained at two frequencies—5 MHz and 20 MHz and the corresponding TFM images—illustrating the AUBT measurement position.

The results presented in Figure 8, Figure 9, Figure 10, Figure 11 and Figure 12 indicate that AUBT shows the presence of HIC at some of locations. At the AUBT-1 measurement position, which, according to the TFM image, is HIC free, the amplitudes of the noise behind the first reflection are similar, which, according to the AUBT, indicates absence of HIC. The results on other measurement positions show a big difference between 5 MHz and 20 MHz signals; the tail amplitude of the 5 MHz signals is higher with respect to the tail amplitude at 20 MHz. In contrast to AVR measurements, the AUBT technique seems to be capable of discriminating HIC defects. For example, at the coinciding measurement point (AUBT-1 and AVR-1), both AVR and AUBT were absent of HIC. In the case of the AUBT 2 and AVR-4 measurement point, HIC was detected by AUBT only.

### 4.4. TULA Method

TULA (TOFD Ultra Low Angle) is time-of-flight diffraction testing (TOFD) performed with ultralow angle ultrasonic transducers. This technique is well suited for the initial screening of a thicker base material. Like TOFD, increased backscattering and clustering in A-Scan signals indicate the HTHA. The TULA method has been proven experimentally as a rather simple but highly sensitive method of detecting small defects such as HTHA or HIC. The scan speed of this method is much higher that of the PAUT method. For this method, special ultrasonic transducers with particular focal depths are required to optimise detection throughout a range of different thicknesses.

**Figure 13:**
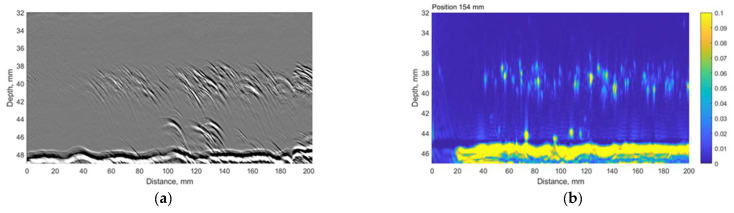
The comparison between (**a**) TULA and (**b**) TFM measurements at 154 mm circumferential scan position. Vertical colour bar represents the peak-to-peak amplitude in arbitrary units.

**Figure 14:**
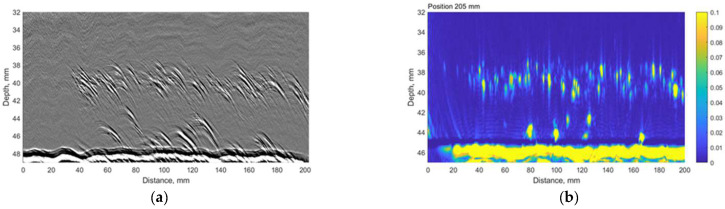
The comparison between (**a**) TULA and (**b**) TFM measurements at 205 mm circumferential scan position. Vertical colour bar represents the peak-to-peak amplitude in arbitrary units.

**Figure 15:**
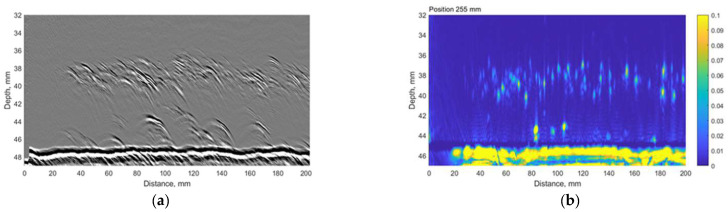
The comparison between (**a**) TULA and (**b**) TFM measurements at 255 mm circumferential scan position. Vertical colour bar represents the peak-to-peak amplitude in arbitrary units.

**Figure 16:**
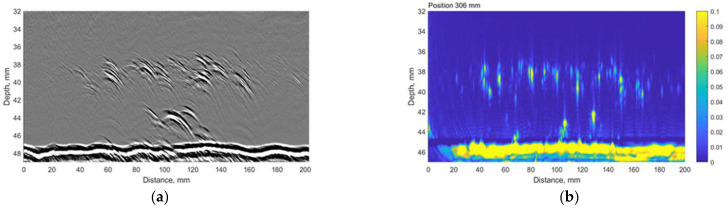
The comparison between (**a**) TULA and (**b**) TFM measurements at 306 mm circumferential scan position. Vertical colour bar represents the peak-to-peak amplitude in arbitrary units.

**Figure 17:**
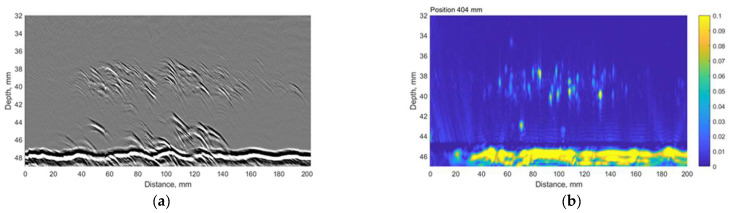
The comparison between (**a**) TULA and (**b**) TFM measurements at 404 mm circumferential scan position. Vertical colour bar represents the peak-to-peak amplitude in arbitrary units.

The time-of-flight diffraction (TOFD) experiments were carried out using Eddify Gekko flaw detector (64:64PR) and the TULA A 10 MHz probe with a 0° roof angle, manufactured by GB Inspection Systems Ltd., Burntwood, UK. The probe was used to scan sample no. 4 at five different circumferential positions. At each circumferential increment, TULA probes were scanned 200 mm along the axial location with the step of 0.12 mm. The probes were driven by 100 V pulse, and the signals were sampled at 100 MHz. The system gain was set to 15 dB. The comparison between the TULA and TFM measurements at circumferential locations summarised in Table 6 are presented in Figure 13, Figure 14, Figure 15, Figure 16 and Figure 17.

A good correlation can be observed between results obtained using the TFM and TULA method. Of course, there are some differences because it is impossible to scan exactly along the same line, especially considering the relatively small dimensions of the defect. The essentially higher resolution of TFM images can be noticed and it can be stated that the TULA technique, in general, enables the detection of HIC damage and is faster and simpler compared to the application of TFM with scanning. It is necessary to notice also that the frequency of TULA transducers (10MHz) is slightly higher that the frequency of the phased array (7.5 MHz) used by the TFM technique.

## 5. Metallographical Examination of a Heat Exchanger Shell Sample

To prove the existence of HIC damage, a metallographic analysis must be performed. In this section, we continue our investigation of two of the most promising techniques, TFM and TULA, on sample no. 5, which is characterised by a metallographic analysis. To demonstrate HIC detection capabilities, TFM and TULA data have been acquired close to the edge of sample no. 5. Then, the sample was sliced, and an examination of the pipe was performed at TFM and TULA measurement locations. Hence, this section first describes the TFM and TULA data acquisition, the results of metallographic analysis and then is finished with a comparative analysis between TFM and metallography.

### 5.1. TFM on Sample No. 5

**Figure 18:**
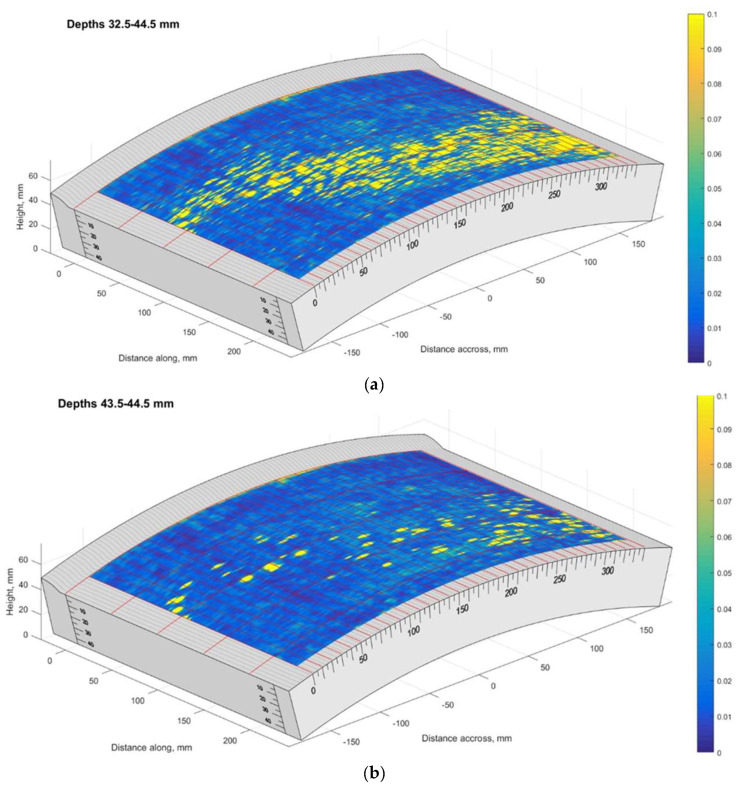
The TFM scan of the Sample No. 5: (**a**) depth range of 32.5–44.5 mm, (**b**) depth range of 43.5–44.5 mm. Vertical colour bar represents the peak-to-peak amplitude in arbitrary units.

**Figure 19:**
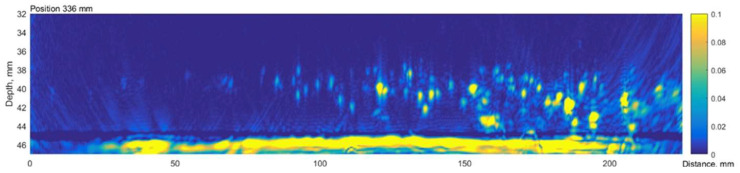
B-scan of the sample No.5 along the edge obtained by the TFM. The B scan corresponds to the section line, where the pipe was sliced before the metallographic examination. Vertical colour bar represents the peak-to-peak amplitude in arbitrary units.

The TFM cartography of sample no. 5 is presented in Figure 18. It can be seen that regions further from the weld were more affected, while the defect distribution is non-uniform. The last TFM scan line according to the circumference of the pipe corresponded to the pipe cut line used for the metallographic analysis. The B-scan of the last circumferential scan position is presented in Figure 19.

The results presented above show that at a distance of 0–50 mm from the weld there are no indications at all; however, from 60 till 210 mm, the B-scan shows a significant amount of reflections located at depths from 35 mm till 44 mm.

### 5.2. Metallographic Examination of Sample 5

**Figure 20:**
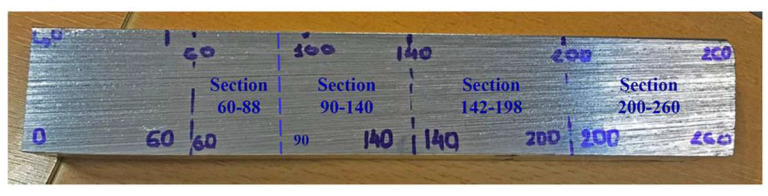
Examined section’s general view.

Metallographic examination of a microstructure was performed in the DEKRA laboratory to verify the ultrasonic testing results. The slice was divided in four sections (60–800 mm, 90–140 mm, 142–198 mm and 200–260 mm) as presented in Figure 20. In the following paragraphs, metallographic images of each section are presented.

**Results on section No1: 60–88 mm**

**Figure 21:**
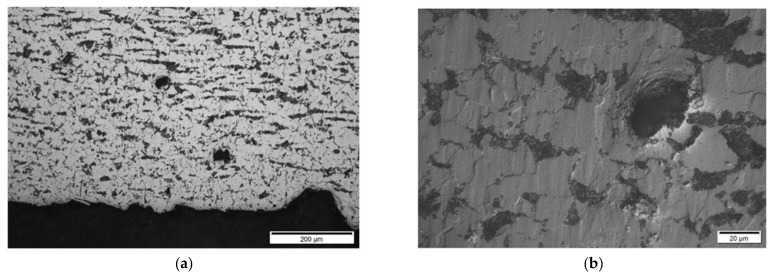
Section No1: 60–88. Coordinate 70 mm, isolated HIC blisters, micro deformation in discontinuity area, magnification 200× (**a**), 1000× (**b**).

Only isolated HIC blisters were revealed at coordinates 70 mm area (see Figure 21). Discontinuities are located near the internal diameter (ID) surface (max. 0.4 mm from ID).

**Results on section No2: 90–140 mm**

**Figure 22:**
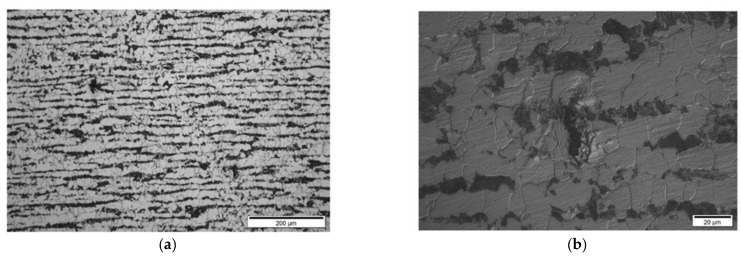
Section No2: 90–140. Coordinate 120 mm, 6.2 mm from ID. Isolated HIC blister, magnification 200× (**a**), 1000× (**b**).

**Figure 23:**
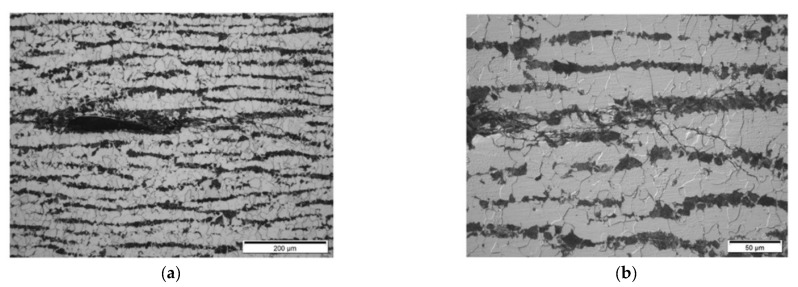
Section No2: 90–140. Coordinate 135 mm, 6.8 mm from ID, blistering cracking in pearlite band, magnification 200× (**a**), 500× (**b**).

**Figure 24:**
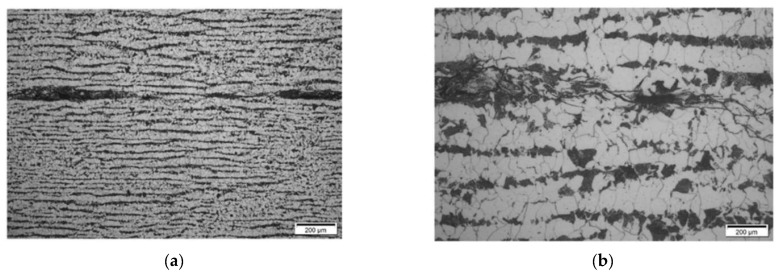
Section No2: 90–140. Coordinate 130 mm, 8.8 mm from ID, straight cracking in pearlite bands, magnification 100× (**a**), 500× (**b**).

Hydrogen-induced damages such as hydrogen-induced cracking, stepwise cracking, straight cracking and blistering cracking, starting from the coordinates 120 mm onwards, were observed (Figure 22, Figure 23 and Figure 24). The maximum depth of defects location in this section is 8.8 mm from ID (37.2 mm from the outer diameter OD, e.g., UT scanning surface). Decarbonised traces originating from the shell ID are clearly seen in the section of thesteel microstructure.

**Results on section No3: 142–198 mm**

**Figure 25:**
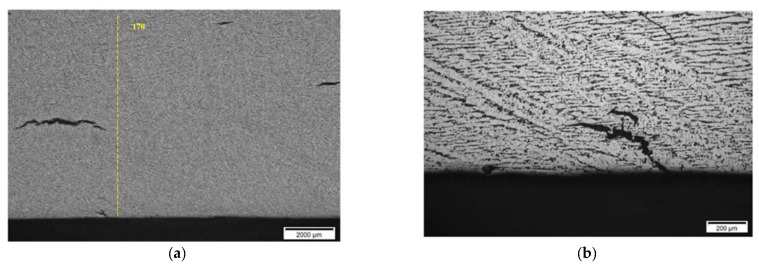
Section No3: 142–198. Coordinate 170 mm. Magnification 12.5× (**a**), Cracking near ID surface, magnification 100× (**b**).

**Figure 26:**
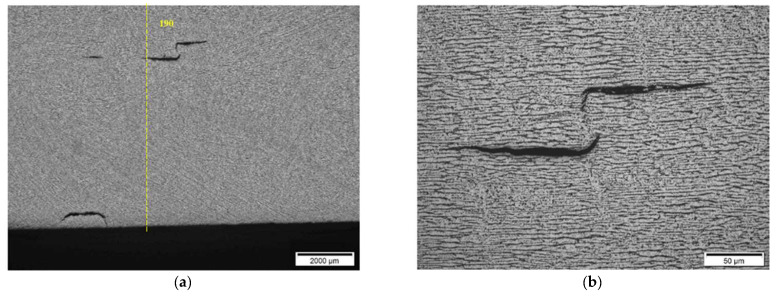
Section No3: 142–198. Coordinate 190 mm. Magnification 12.5× (**a**), 6.1–6.5 mm from ID, stepwise cracking magnification 50× (**b**).

**Figure 27:**
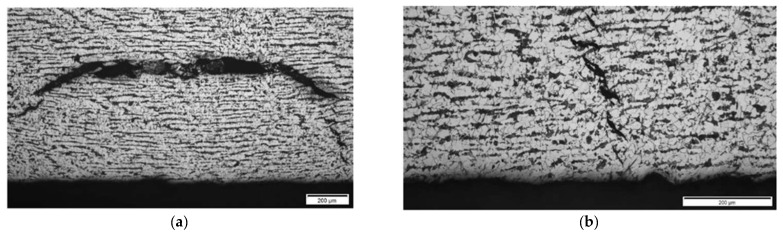
Section No3: 142–198. Coordinate 193 mm, blistering cracking magnification ×100 (**a**), stepwise cracking connected to ID, magnification 200× (**b**).

Hydrogen-induced damage such as hydrogen-induced cracking, stepwise cracking, straight cracking, blistering cracking were observed (Figure 25, Figure 26 and Figure 27). The maximum depth of defects location in this section is 8.8 mm from ID (37.2 mm from OD). Decarbonised traces originating from shell ID are also clearly seen in the section of the steel microstructure.

**Results on section No4: 200–260 mm**

**Figure 28:**
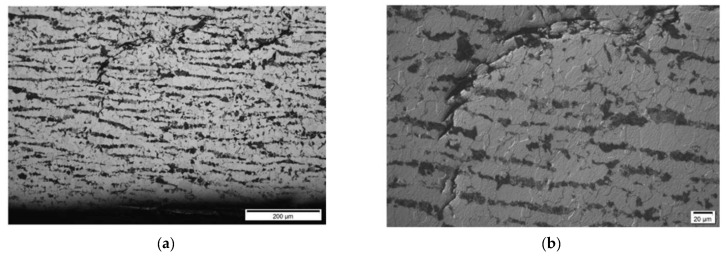
Section No4: 142–198. Coordinate 190 mm. Magnification 12.5× (**a**), 6.1–6.5 mm from ID, stepwise cracking magnification 50× (**b**).

**Figure 29:**
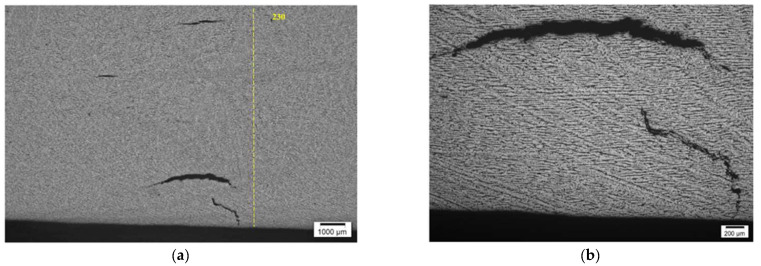
Section No4: 142–198. Coordinate 190 mm. Magnification 12.5× (**a**), 6.1–6.5 mm from ID, stepwise cracking magnification 50× (**b**).

Hydrogen-induced damage, as mentioned above, such as hydrogen induced cracking, stepwise cracking, straight cracking, blistering cracking, were observed (Figure 28 and Figure 29). The maximum depth of defects location in this section is 8.0 mm from ID (or 38.0 mm from OD (UT scanning surface)). Decarbonised traces originating from the shell ID are also clearly seen in the of the section steel microstructure.

## 6. Discussion

A comparison of metallographic images (Figure 21, Figure 22, Figure 23, Figure 24, Figure 25, Figure 26, Figure 27, Figure 28 and Figure 29) with the results obtained using TFM (Figure 19) revealed a good correlation. In the first section (distances 60–88 mm), TFM images did not show any indications. At the same time, metallographic analysis found only a minor number of small blisters with dimensions around 20 µm. Such size defects are not visible for ultrasonic inspection at frequencies used in this study. An additional problem is caused by the fact that they are situated very close to the wall of the inner surface and cannot be indicated by any of the used techniques due to strong backwall reflection. 

In section No2 (distances 90–140 mm), metallography shows multiple defects with dimensions 20 µm to 200 µm. The TFM data also show multiple, relatively low amplitude indications. So, it can be stated that the TFM method enabled the detection of HIC damage when a single crack reaches dimensions close to 200 µm. As a result of the good correlation between TFM and TULA measurements, as shown in the previous section, it was expected that TULA could also successfully detect these indications.

In section No3 (distances 142–198 mm), multiple cracks reaching sizes over 2 mm can be observed, which are indicated in TFM images by a strong concentrated reflection. Such a level of HIC damage can easily be detected by TFM (Figure 19).

In the last analysed section No4 (distances 200–260 mm), cracks with sizes up to 2 mm are detected by metallography, correspondingly indicated in the TFM images, although the image is not so sharp. It can be noticed that in TFM image, the back wall reflection is almost absent, but indications of a crack are present. This is due to the fact that this section is at the edge of the sample and the phased array was not fully scanned in this zone. On the other hand, TFM reconstruction was performed not only in the zone situated directly under the phased array, but in an area in the front direction from the edge of the array. So, the results presented demonstrate that this technique can be used even in partially accessible zones.

While the performance of the AVR and AUBT techniques for HIC detection was found to be similar to that reported elsewhere, the results obtained with the TFM and TULA techniques introduced some positive aspects to the current state-of-the-art methods. Even though phased array measurements are included in recommended practices, the TFM method allows one to significantly increase the resolution and to detect cracks at earlier stages. It has been demonstrated that TFM can detect stage 2 HTHA with a crack size of 300 µm [23], while the results obtained in this paper support such statements. On the other hand, the results using TULA probes appear to be quite novel and demonstrate that such a technique can be used with similar confidence as TFM. Since the TULA technique requires a two-channel ultrasonic system, it can be considered cheap and may be used for the primary detection of hydrogen-induced cracking. Meanwhile, TFM can be used for a more precise assessment of areas potentially affected by HIC.

In general, it can be noticed that most authors investigate NDT techniques as methods for the detection of single defects of a particular size, as well to characterise them. In this case, dimensions of the defects play the main role; in other words, they determine how small defects of a particular type are detectable. However, in the case of the assessment of early stages of HIC or HTHA, the cavities and micro-cracks are so small that detecting them pushes ultrasonic methods to their limit. On the other hand, hydrogen-induced damage is not a single but is a multiple set of scattered cracks in an area as a result of changes in the metal structure, which then later develop into larger cracks. In the presented study, the main accent was put not on the characterisation of a singular small defect, but to detect and assess the areas affected by hydrogen-induced cracking. In such a case, only the TULA and TFM methods have demonstrated the possibility of practical applications.

## 7. Conclusions

1. It was found that the totally focused method is the most effective for the detection of HIC in different stages of development and enables at 7.5 MHz the detection of blisters and cracking with dimensions of 200 μm. Application of additional scanning along both axes and the merging of the images makes it possible to map the damaged areas.
2. The TULA technique also demonstrated the possibility of detecting zones with a lot of small reflectors, a characteristic for HIC affected zones. Hence, the TULA method can be used as a fast technique for the detection of HIC. However, a comparison of TFM and TULA methods showed that the spatial resolution of the TFM is higher.
3. The advanced ultrasonic backscatter technique based on the analysis of the ultrasonic signals backscattered by fissures demonstrated good performance and can be recommended as an additional tool for the assessment HIC at positions with already detected indications. It is not practical in scanning, as the measurements should be performed at exactly the same position, otherwise the results will be ambiguous.
4. The technique based on the assessment of velocity ratio does not demonstrate good performance in the case of thick-walled analysed samples. This technique is probably applicable when the component is damaged by HIC in the entire thickness of it. Otherwise, the sensitivity reduces depending on the ratio of the damaged area thickness to the total thickness of the component.

## Figures and Tables

**Table 1:** Elemental composition of sample no. 4 obtained using X-ray fluorescence spectrometer.

Element Symbol	Percentage (%)	+/−3σ
Fe	97.06	0.16
Mn	1.112	0.083
Si	0.94	0.11
S	0.354	0.037
Cu	0.244	0.049
Ni	0.159	0.044
Ti	0.053	0.046
P	0.029	0.033
Cr	0.025	0.020
Nb	0.021	0.005

**Table 2:** Circumferential and axial scan lengths for each test piece under inspection.

Test Piece Reference No.	Parameter	Value
Test piece no. 4	Axial scan length	400 mm
	Circumferential scan length	462 mm
Test piece no. 5	Axial scan length	165 mm
	Circumferential scan length	339 mm

**Table 3:** The phased array, wedge, pulser, scanner and reconstruction parameters used in the experiments.

Parameter	Value	Parameter Group
Array frequency	7.5 MHz	Phased array
Number of elements	64	
Array pitch	0.5 mm	
Interelement spacing	0.1 mm	
Active length	31.9 mm	
Elevation	9 mm	
Bandwidth at −6 dB	≥55%	
Wedge material	Plexiglas	Wedge
Wedge dimensions	37 mm × 73 mm × 20 mm	
(width × length × thickness)		
Longitudinal wave velocity	2700 m/s	
Excitation	100 V bipolar square pulse	Pulser/receiver
Sampling frequency	100 MHz	
Axial scan step	1 mm	Scanner
Circumferential scan step	3 mm	
Encoder resolution (axial scan)	16 pts/mm	
TFM reconstruction zonebreak//(width × height)	60 mm × 45 mm	TFM reconstruction
Number of pixels	94 k	
Pixel size	0.17 mm (λ/46)	

**Table 4:** Coordinates of acquisition points for AVR measurements.

Measurement Point Reference	Circumferential Position (mm)	Axial Position (mm)
AVR-1	154	20
AVR-2	154	70
AVR-3	205	20
AVR-4	205	120
AVR-5	454	120

**Table 5:** Coordinates of acquisition points for AUBT measurements.

Measurement Point Reference	Circumferential Position (mm)	Axial Position (mm)
AUBT-1	154	20
AUBT-2	205	120
AUBT-3	255	70
AUBT-4	306	120
AUBT-5	404	120

**Table 6:** Coordinates of acquisition points for TULA measurements.

Measurement Point Reference	Circumferential Position (mm)
TULA-1	154
TULA-2	205
TULA-3	255
TULA-4	306
TULA-5	404

## Data Availability

Not applicable.
